# Present and future of microbiome-targeting therapeutics

**DOI:** 10.1172/JCI184323

**Published:** 2025-06-02

**Authors:** Lauren E. Lynch, Rachel Lahowetz, Christian Maresso, Austen Terwilliger, Jason Pizzini, Valeria Melendez Hebib, Robert A. Britton, Anthony W. Maresso, Geoffrey A. Preidis

**Affiliations:** 1 Division of Gastroenterology, Hepatology and Nutrition, Department of Pediatrics, Baylor College of Medicine and Texas Children’s Hospital, Houston, Texas, USA.; 2 Department of Molecular Virology and Microbiology,; 3 TAILOR Labs, and; 4 Alkek Center for Metagenomics and Microbiome Research, Baylor College of Medicine, Houston, Texas, USA.; 5 USDA/ARS Children’s Nutrition Research Center, Houston, Texas, USA.

## Abstract

A large body of evidence suggests that single- and multiple-strain probiotics and synbiotics could have roles in the management of specific gastrointestinal disorders. However, ongoing concerns regarding the quality and heterogeneity of the clinical data, safety in vulnerable populations, and the lack of regulation of products containing live microbes are barriers to widespread clinical use. Safety and regulatory issues must be addressed and new technologies considered. One alternative future strategy is the use of synthetic bacterial communities, defined as manually assembled consortia of two or more bacteria originally derived from the human gastrointestinal tract. Synthetic bacterial communities can model functional, ecological, and structural aspects of native communities within the gastrointestinal tract, occupying varying nutritional niches and providing the host with a stable, robust, and diverse gut microbiota that can prevent pathobiont colonization by way of colonization resistance. Alternatively, phage therapy is the use of lytic phage to treat bacterial infections. The rise of antimicrobial resistance has led to renewed interest in phage therapy, and the high specificity of phages for their hosts has spurred interest in using phage-based approaches to precisely modulate the microbiome. In this Review, we consider the present and future of microbiome-targeting therapies, with a special focus on early-life applications, such as prevention of necrotizing enterocolitis.

## Introduction

It is intriguing to consider the notion of improving health via therapeutics that target the trillions of microbes residing within the human gut (1). However, despite a multi-billion dollar “gut health” supplement industry and thousands of published clinical trials to date, overall evidence is limited that microbiome-targeting therapies affect clinically relevant outcomes. Guidelines issued in 2024 by the American Gastroenterological Association (AGA) include conditional recommendations suggesting that fecal microbiota–based therapies may be useful for select subsets of adults with recurrent, severe, or fulminant *Clostridioides difficile* infection, but these recommendations are based on low or very low certainty of evidence (2). The AGA separately published clinical practice guidelines suggesting potential uses of specific probiotics (also with low or very low certainty of evidence) to prevent *C*. *difficile* infection among adults and children receiving antibiotics and to prevent pouchitis among patients with ileal pouch–anal anastomosis resulting from chronic ulcerative colitis (3). There was too little evidence to make any recommendation regarding probiotics for patients with *C*. *difficile* infection, Crohn’s disease, ulcerative colitis, or irritable bowel syndrome, and the AGA recommended against giving probiotics to children with acute infectious gastroenteritis. In contrast, there was moderate to high certainty of evidence suggesting that specific probiotics given to preterm, low-birth-weight infants may reduce the risk of necrotizing enterocolitis (NEC), a devastating disease of prematurity (3). This conditional recommendation remains the strongest vote of confidence in any microbiome-targeting strategy by a major clinical society to date. Despite the substantial evidence base, probiotics are not being offered routinely for newborns or to any other patient population. This Review considers present and future applications of microbiome-targeting therapies, with a special focus on the newborn period, when therapeutic manipulation of gut microbes could be most impactful.

## The newborn gut microbiome in health and disease

Healthy full-term infants enter the world with a sterile intestine that is colonized by microbes from the maternal gut, birth canal, skin, and milk. These bacteria follow predictable patterns of succession (4, 5). Unlike adult gut microbial communities, which are complex and stable over time, the infant microbiota is less diverse and undergoes profound phylum-level changes in early life (6). Decreased diversity is due in part to uneven microbial communities that are dominated by bacteria that preferentially consume human milk oligosaccharides (HMOs), a major component of the infant diet. In prematurity, normal patterns of microbial colonization and succession are disrupted by factors including cesarean delivery, delayed maternal contact, feeding practices including parenteral nutrition, and routine use of broad-spectrum antibiotics to “rule out” early-onset sepsis. Early gut colonizers include microbes endemic to the neonatal intensive care unit (NICU) environment (7). Preterm infants exhibit even lower microbial community diversity than full-term infants, with as few as a dozen taxa present in the second month of life (8). Whereas the gut microbiota of full-term neonates is composed predominantly of HMO-consuming species within the genera *Bifidobacterium* and *Bacteroides*, preterm infants harbor greater abundances of potentially pathogenic taxa within the phyla Proteobacteria and Firmicutes with concurrent decreases in Bacteroidetes. These community structural changes could be clinically relevant, given the observation that increased Proteobacteria abundance often precedes the onset of NEC (9–11).

**Figure 1:**
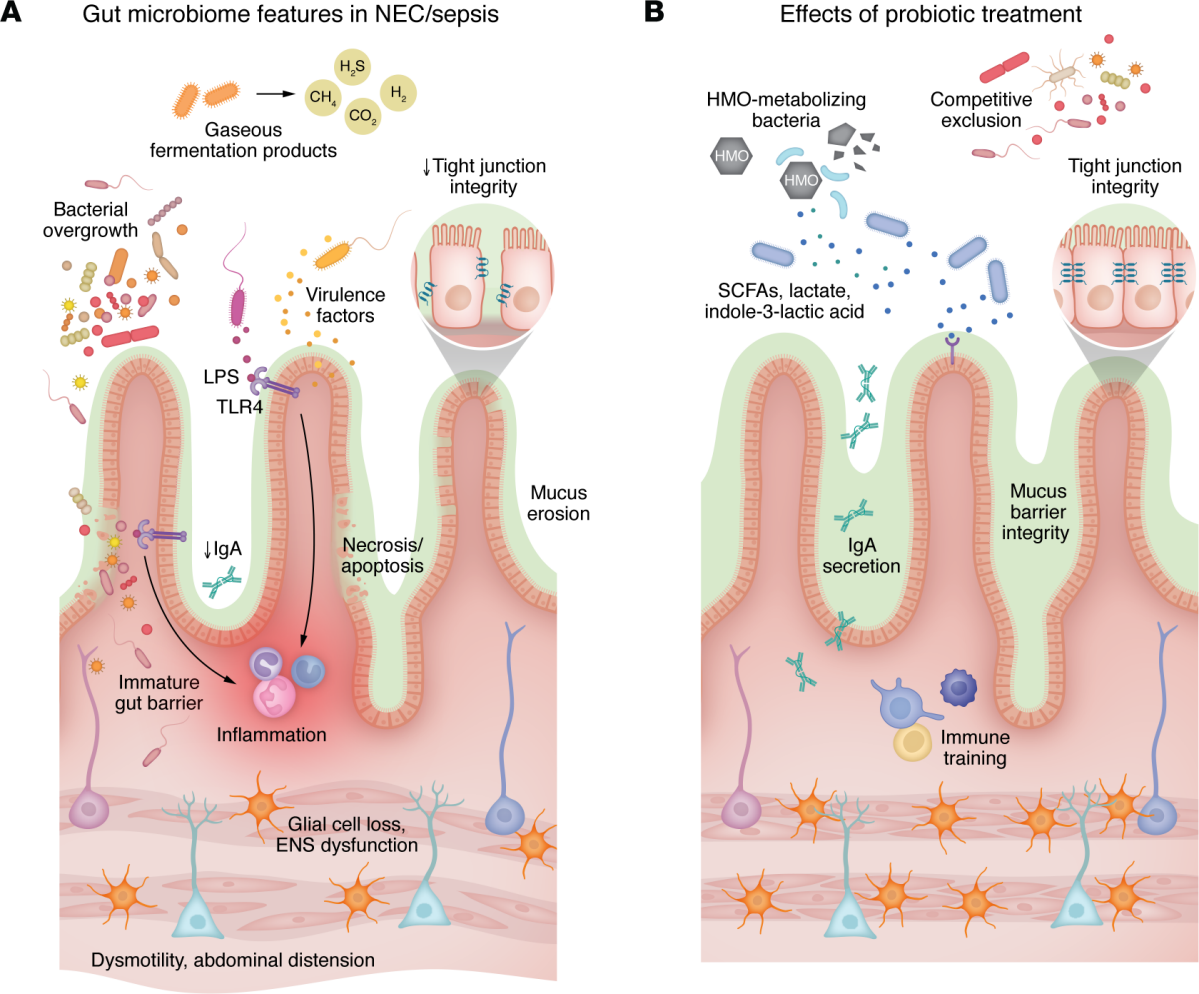
Pathophysiology of NEC and sepsis and potential probiotic effects. (**A**) Pathological features of NEC and sepsis include bacterial overgrowth, microbial virulence factors and gaseous fermentation products, and uncontrolled inflammation. Dysregulated TLR4 signaling due to lipopolysaccharide and insufficient mucosal IgA production threatens the integrity of the immature gut barrier and causes enteric nervous system (ENS) dysfunction resulting in dysmotility and abdominal distension. (**B**) Probiotics, including strains that metabolize human milk oligosaccharides (HMOs), can produce short-chain fatty acids (SCFAs), lactate, and indole-3-lactic acid. These metabolites may strengthen the intestinal barrier by enhancing mucus production, IgA secretion, tight junction integrity, and immune training. Competitive exclusion of pathogens further protects against NEC and sepsis.

NEC affects 6%–7% of newborns weighing less than 1,500 g and typically presents in the second week of life with feeding intolerance, abdominal distention, and bloody stools. Radiographic findings may include portal venous gas or intramural gas, known as pneumatosis intestinalis. In severe cases, necrotic bowel requires surgical resection, resulting in short-bowel syndrome and long-term dependence on parenteral nutrition. Mortality rates approximate 20%–30% (12). The pathophysiology of NEC remains poorly understood. Recent evidence implicated uncontrolled activation of the innate immune receptor TLR4 in the intestinal epithelium in response to an altered microbiota in a fragile newborn with an underdeveloped gut barrier and immature mucosal immunity (Figure 1A). Impaired proliferation and healing observed in the newborn’s intestinal epithelial cell population caused irreversible injury associated with cell death by apoptosis, autophagy, and necrosis (13). In a mouse model of NEC, excess TLR4 signaling also caused enteric glial loss manifesting as intestinal dysmotility, abdominal distention, and feeding intolerance (14). Severe cases of NEC invariably lead to sepsis, disseminated infection, and multiorgan dysfunction, although sepsis may precede or contribute to NEC pathogenesis (15–17). Despite numerous studies, there is limited consensus on specific bacterial species or metabolites as reliable biomarkers for NEC onset. Further research is essential to better stratify preterm infants and identify those most likely to benefit from targeted microbiome-based interventions.

The working hypothesis that abnormal intestinal colonization is the inciting factor for inflammation, NEC, and sepsis provides a strong rationale for therapeutic microbiome modulation in the newborn period. Microbiome-targeting strategies, mainly single- and multiple-strain probiotics, have been studied extensively in newborns. Despite evidence that probiotics may reduce morbidity and mortality, further steps are needed before routine administration to newborns becomes standard of care in the United States.

## Current state of microbiome-targeting therapies for newborns

Four decades of clinical investigation into probiotics for newborns has produced a substantial body of evidence. The total number of preterm infants enrolled in probiotic randomized clinical trials (RCTs) exceeds the number of participants in RCTs testing probiotics for any other gastrointestinal disorder (18). One systematic review and network meta-analysis identified 63 RCTs with 15,712 preterm, low-birth-weight infants (19). Seven probiotic strains or combinations reduced the risk of severe NEC, but the quality of evidence varied among treatments. Among the interventions with moderate- or high-quality evidence, a combination of one or more *Lactobacillus* spp. and one or more *Bifidobacterium* spp. reduced both the incidence of severe NEC (OR, 0.35; 95% CI, 0.20–0.59) and all-cause mortality (OR, 0.56; 95% CI, 0.39–0.80). Although this combination ranked highest among single- and other multiple-strain probiotics, this was the most frequently tested treatment (11 RCTs, 1,878 infants), not necessarily the treatment with the greatest effect size. Despite theoretical concerns that live microbes may translocate the gut barrier and cause sepsis, trends toward reduced odds of culture-proven sepsis were observed for most probiotics. The body of evidence was large but imperfect: only 22 of the 63 studies showed low risk of bias in all six domains assessed and were free of industry funding (19).

These results are congruent with a systematic review in the Cochrane Database that included 60 RCTs with 11,156 very preterm or very low-birth-weight infants (20). Standard meta-analysis concluded that overall, probiotics may reduce the risk of NEC (risk ratio [RR], 0.54; 95% CI, 0.46–0.65), with a number needed to treat for an additional beneficial outcome (NNTB, a measure of treatment effect size in which lower value indicates greater efficacy) of 33 (95% CI, 25–50; low certainty), and probably reduce all-cause mortality (RR, 0.77; 95% CI 0.66–0.90), with a NNTB of 50 (95% CI, 50–100; moderate certainty). This analysis highlighted the limited data available for the subpopulation of extremely preterm (<28 weeks gestation) or extremely low-birth-weight (ELBW, <1,000 g) newborns. In this subpopulation, probiotics may have little or no effect on NEC (RR, 0.92; 95% CI, 0.69–1.22; 10 trials; 1,836 infants; low certainty) or all-cause mortality (RR, 0.92; 95% CI, 0.72–1.18; 7 trials; 1,723 infants; low certainty) (20). Thus, the risk-benefit ratio seems less favorable for ELBW neonates.

Another systematic review identified 106 RCTs with 25,840 infants assessing probiotics, prebiotics, lactoferrin, and combinations thereof (21). Network meta-analysis found moderate- to high-certainty evidence that multiple-strain probiotics reduce severe NEC (RR, 0.38; 95% CI, 0.30–0.50) and all-cause mortality (RR, 0.69; 95% CI, 0.56–0.86). Similar concerns regarding risk of bias were noted. Specifically, 25 RCTs had a high risk of bias, as outcome assessors might have been aware of which treatment group participants were in, and 48 RCTs had a high risk of bias for masking of outcome assessments (21). Another important RCT studied a synbiotic: Among 4,556 full-term and late-preterm newborns in rural India, 10^9^ *Lactiplantibacillus plantarum* ATCC 202195 and 150 mg fructooligosaccharide per day reduced the combined outcome of sepsis and death (RR, 0.60; 95% CI, 0.48–0.74) compared with placebo (22). Thus, microbiome-targeting therapies show promise in both developed and resource-constrained settings.

This body of evidence formed the basis for guidelines issued by the AGA (moderate/high certainty) (3), the European Society for Pediatric Gastroenterology, Hepatology and Nutrition (low certainty) (23), and the World Health Organization (moderate certainty) (24). Each organization conditionally recommended that probiotics be offered for preterm or low-birth-weight infants. However, a commentary from the American Academy of Pediatrics recommended against routine probiotic use in NICUs, citing concerns including the overall strength, quality, and heterogeneity of the clinical data and the potential of probiotics to do harm in vulnerable populations (25). Consequently, the proportion of US NICUs using probiotics decreased from roughly half to just 29%–39% in 2021 (26, 27). In fall 2023, the US FDA issued warning letters to two manufacturers and clinical providers after an infant with ELBW who had been receiving a probiotic developed sepsis and subsequently died. The bacterium associated with the sepsis was a genomic match to the probiotic *Bifidobacterium longum* that was administered, although the cause of death in this case remains unclear. The FDA reminded manufacturers that marketing unapproved new drugs and unlicensed biological products for “the mitigation, treatment, or prevention of disease” is unlawful (28, 29) and reminded clinicians that administering probiotics with this intent requires an Investigational New Drug (IND) application to be filed with the FDA (30). The response was swift; nearly all NICUs in the United States ceased offering probiotics to newborns and continue this policy today (27). Given the strongly worded FDA warnings and the litigious nature of neonatal care in the United States, it is unlikely that most NICUs will resume routine use of unapproved, unlicensed probiotic products. Because the IND pathway is time- and resource-intensive, new strategies must be considered. Importantly, a scientific basis for selecting optimal microbiome-based therapies must be established.

## Future strategy 1: pursuing clinical impact with probiotics

The most direct path to clinical impact for microbiome-targeting therapies involves addressing three concerns regarding probiotic use. The first relates to the quality and strength of the evidence. The authors of the Cochrane Review concluded that “there is a need for further large, high-quality trials to provide evidence of sufficient validity and applicability to inform policy and practice” (20). Indeed, the existing evidence is fraught with risk of bias and with striking study heterogeneity: Most RCTs differ in probiotic strain(s), doses and durations of therapy, and newborn inclusion criteria (e.g., gestational age, birth weight). Thus, it is remarkable that standard meta-analysis finds such a striking effect size: approximately 50% reduction of NEC (20). Large effect sizes are the most common reasons guideline committees rate up the quality of a body of evidence (31). It is unlikely that newer RCTs will substantially influence overall estimates of treatment effect sizes. For this to happen, a hypothetical RCT enrolling 80,000 newborns would need to find probiotics to be equivalent to placebo (32). Furthermore, one of the few RCTs to use stool cultures to quantify cross-contamination reported that despite strict containment protocols, guts were colonized by probiotics in 49% of infants in the placebo arm (33). Thus, the true effect size by which probiotics reduce the risk of NEC relative to placebo may be masked by plausible residual bias. Finally, ethical clinical research requires equipoise, which is only satisfied when the expert medical community is genuinely uncertain of therapeutic merits. Many experts question whether additional placebo-controlled trials should be performed. But with neonates with ELBW, in whom probiotics do not seem to confer the same benefit (20), high-quality studies are warranted. These RCTs should be thoughtfully designed and executed: all protocols should be preregistered, bias potential should be minimized, and reports should adhere to standardized guidelines (34).

Confidence in the existing evidence base would be increased if the probiotic functions responsible for the beneficial effects on NEC, sepsis, and mortality were elucidated. Most RCTs have tested strains of *Lactobacillus* and/or *Bifidobacterium*, not because of well-characterized mechanisms of action in the newborn gut but rather due to historical precedent and prior use in other clinical scenarios. Laboratory models have been used to show that a range of probiotics can strengthen the intestinal barrier, support enteric nervous system development, and promote immune system maturation. Bacterial metabolites, including short-chain fatty acids, lactate, and indole-3-lactic acid formed from the degradation of HMOs, can promote neonatal growth, reduce inflammation, and decrease gut barrier permeability (35, 36). These and other mechanisms might contribute to the protective effects of probiotics in the newborn period (Figure 1B). Better preclinical in vivo models to study the early-life microbiome are needed. Simultaneously, mechanistic insights can be gained from clinical trials. In one illustrative example, metabolomic analysis of infants supplemented with a commercial formulation of *Bifidobacterium bifidum* and *Lactobacillus acidophilus* revealed higher fecal acetate and lactate concentrations and lower fecal pH, consistent with the ability of the administered bifidobacterial strain to metabolize HMOs into acetate (37). Another study analyzed blood, stool, and the human milk diet to reveal evidence of increased HMO consumption (fewer intact HMOs and increased indole-3-lactic acid), along with decreased concentrations of calprotectin as a surrogate marker of inflammation, in infants receiving *Bifidobacterium infantis* EVC001 compared with those receiving probiotic *Limosilactobacillus reuteri* DSM 17938 (38). Not all *Bifidobacterium* genomes encode the glycoside hydrolases that are essential for the degradation of HMOs, underscoring the need for careful strain selection (39). On the other hand, given the phylogenetic diversity of probiotic organisms that confer similar beneficial effects in the newborn gut, one must consider the possibility that probiotic benefits might not be dependent on strain-specific functions. Rather, benefits might be conferred via broader mechanisms, including competitive exclusion in the sparsely populated newborn gut, which has few resident microbes and is susceptible to colonization by pathobionts from the NICU environment.

Second, safety concerns must be addressed. In 2014, an infant born at 29 weeks gestation and 1,400 g died from gastrointestinal mucormycosis, a fungal infection that was traced to contamination during manufacturing of a probiotic administered in the NICU (40, 41). In the absence of FDA oversight, a multipronged strategy has been proposed to minimize the risk of additional deaths due to product contamination (42). This framework involves companies manufacturing products under stringent conditions overseen by independent third parties; hospitals implementing protocols to avoid contamination during storage, preparation, and administration; and providers monitoring for and reporting adverse events to the FDA MedWatch program (43). Even if the possibility of product contamination could be eliminated, there would remain a risk of translocation of live probiotics from the intestine, as might have occurred in the death associated with probiotic *B*. *longum*. This risk is difficult to quantify, because the frequency of invasive infection due to probiotics is unknown. No RCT to date has reported any probiotic sepsis in preterm newborns. However, not all clinical laboratories are equipped to identify probiotic species from clinical samples, and harms reporting in probiotic trials historically has been incomplete (44). A recent systematic review sought to identify all known cases of probiotic sepsis in preterm infants. From 1,569 published studies, 32 cases of probiotic sepsis, including two infant deaths (one mechanistically unrelated), were identified. These events were reported in 15 case reports/series and one non-RCT observational study (45). Thus, the total number of infants who ever received probiotics (the “denominator” in calculating the rate of probiotic sepsis from these 32 cases) is unknown.

A third concern pertains to the lack of approval of probiotics by the FDA or other regulatory authority. Currently, most clinicians in the United States are reluctant to offer probiotics for preterm neonates. Hospital legal teams discourage their use due to the possibility of lawsuits. FDA approval of routine probiotics as live biotherapeutic products may be prerequisite to their use in US hospitals. There is movement in this direction. Two phase III placebo-controlled, multicenter trials are investigating probiotics as INDs under FDA oversight. The Connection Study is testing daily *L*. *reuteri* IBP-9414 in 2,158 infants born at 23–32 weeks gestation and 500–1,500 g — with follow-up until the participant reaches 35 weeks postmenstrual age — in NICUs in the United States and 9 countries in Europe and the Middle East (NCT03978000). And the Probiotic Supplementation in Extremely Preterm Infants in Scandinavia (PEPS) trial (46) is testing the combination of 300 million *B*. *infantis* Bb-02, 300 million *Bifidobacterium lactis* Bb-12, and 350 million *Streptococcus thermophilus* TH4 daily until gestational week 34 in 1,620 extremely preterm infants in 6 NICUs in Sweden and Denmark (NCT05604846). Ultimate approval of either of these products would transform clinical practice in the United States by offering providers the possibility of using, for the first time, a probiotic that has completed the FDA’s rigorous premarket review evaluation for safety and effectiveness.

The restrictive approach taken by the FDA stands in stark contrast to policies adopted by the remainder of the developed world. Canada (47), Europe (23), and New Zealand (48) implemented distinct regulatory frameworks, highlighting potential avenues for policy reform to enable current probiotic formulations to be administered safely in hospitals. A change in FDA policy would be much less expensive than the ongoing live biotherapeutic approval process. Policy change is the most direct path to resuming newborns’ access to potentially life-saving probiotic therapies.

## Future strategy 2: synthetic bacterial communities

Rationally selected microbiome-targeting therapies that support newborn gut development and function could facilitate more effective preventions for NEC and sepsis. One future strategy is the use of synthetic bacterial communities, defined as manually assembled consortia of two or more bacteria originally derived from the human gastrointestinal tract. In reference to a full-term infant, preterm infants undergo aberrant acquisition and assembly of the gut microbiome, predisposing them to suboptimal health outcomes. Providing these infants with a robust gut microbiome in early life in lieu of allowing aberrant colonization with NICU-endemic bacteria may significantly improve predisposition to NEC and sepsis. Synthetic bacterial communities can model functional, ecological, and structural aspects of native communities within the gastrointestinal tract (49), occupying varying nutritional niches and providing the infant with a stable, robust, and diverse gut microbiota that can prevent pathobiont colonization by way of colonization resistance (50). This consortium of bacteria also might be able to benefit infants beyond colonization resistance through cometabolism of host-derived molecules, fermentation of dietary substrates, or immune training.

**Figure 2:**
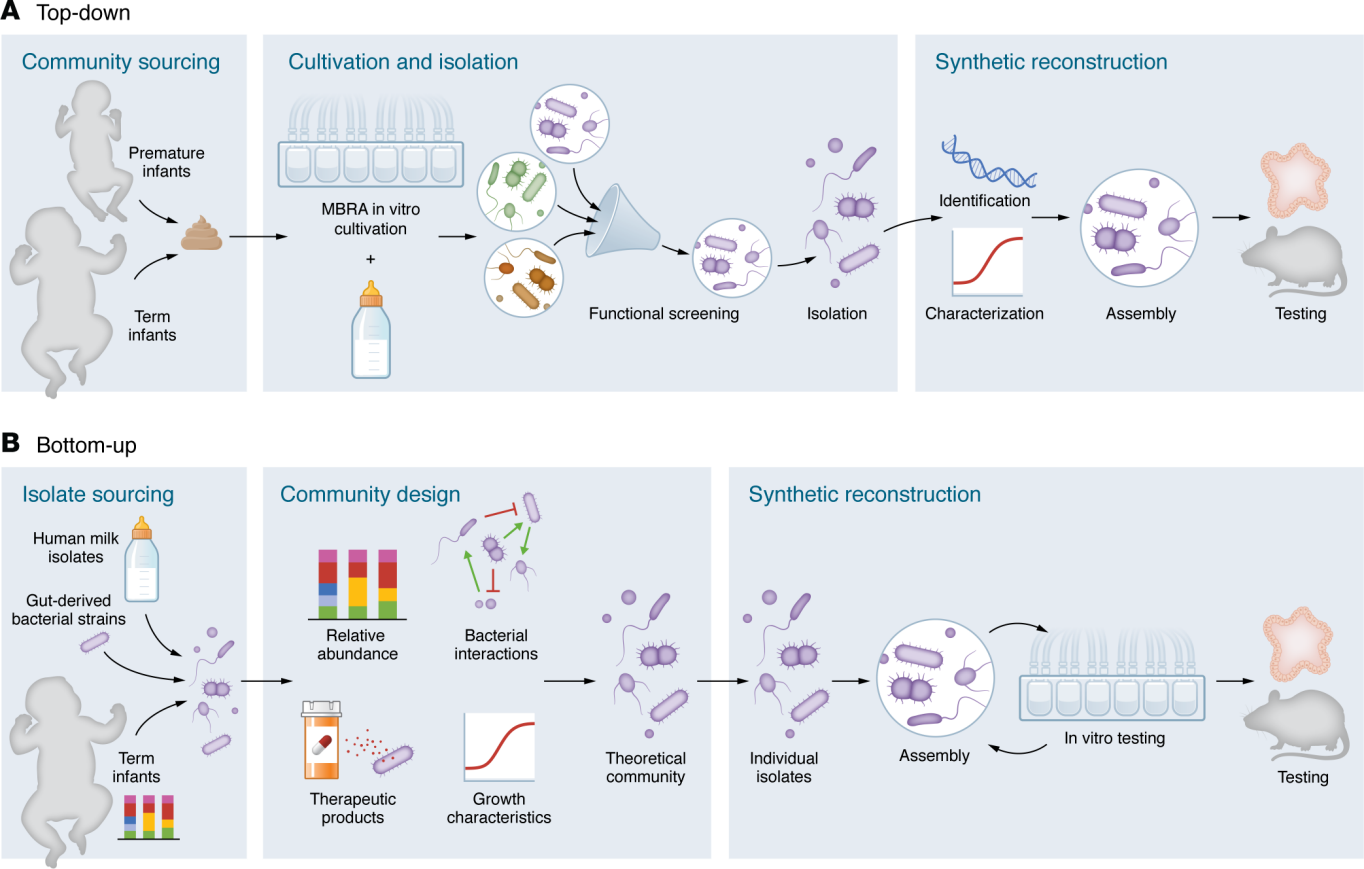
Generation of synthetic bacterial communities. Overview of (**A**) top-down and (**B**) bottom-up strategies to design, assemble, and test synthetic bacterial communities for application in preterm infants. MBRA, minibioreactor array.

Synthetic bacterial communities may be designed and assembled using a top-down approach (Figure 2A) (51) in which environmentally established microbial communities are simplified while retaining desired functional outputs. One strategy uses human stool to inoculate in vitro gut model systems such as minibioreactor arrays, which facilitate continuous inflow of media and outflow of waste to create stable bacterial communities (52). In vitro cultivation enables the selection of microbial communities that confer colonization resistance or produce favorable metabolic byproducts (53, 54). Members of the resulting communities can be identified by sequencing, isolated as pure strains, and characterized. Synthetic communities can be assembled based on the identity and distribution of bacteria within the native community. Top-down approaches have the advantage of leveraging the existing interactions and known stability of natural communities, allowing for the control and reproducibility of synthetic derivatives. Limitations of top-down approaches include media selection bias, in which key community members might be lost in vitro, and the accrual of genomic changes in the cultivation process, which could confer loss of functional capacity, community instability, or antibiotic resistance (55).

Alternatively, a bottom-up approach leverages existing knowledge of metagenomics, abundances within the gut, and growth parameters of candidate microbes to build synthetic bacterial communities that achieve specific functional goals (Figure 2B) (49). In one example, an 11-member community was designed by Van der Lelie and colleagues (56) to confer metagenomic functions depleted in patients with inflammatory bowel disease (IBD). Community member selection was based on in silico predictions of bacterial interactions and therapeutic products with antiinflammatory and antimicrobial properties. In a humanized mouse model of chronic T cell–mediated IBD, this synthetic bacterial community decreased pathobiont abundance, reduced inflammation, and reversed colitis (56). Designing synthetic communities similar to this 11-member community that are intended to reduce intestinal inflammation and to competitively exclude pathobionts represents a potential approach to preventing NEC and sepsis. However, limitations of the bottom-up approach include uncertainties about successful engraftment within the host, the inability to incorporate unculturable species, and potential discrepancies between in silico predictions and actual species interactions mediated by cellular contact, quorum sensing, or metabolite production (49). Moreover, synthetic communities may overlook key interactions with other microbial communities, including fungi and viruses, which could influence colonization success and function.

Developing synthetic bacterial communities specifically for infants requires several considerations. These include determining the most appropriate donor source of fecal communities and selecting members of the native community for inclusion in the synthetic community. Since preterm infants (even those who do not develop NEC or sepsis) are haphazardly colonized by NICU-endemic microbes and do not exhibit typical patterns of microbiome maturation (8, 57), bacteria could be derived from healthy full-term infants. However, whether engraftment of full-term infant–derived microbes would occur efficiently in the premature intestine is uncertain. Another source to consider for this approach is human milk, which is not only the primary neonatal diet but a major source of neonatal gut colonization (58, 59). Le Bras and colleagues (60) demonstrated that two 11-member synthetic bacterial communities assembled from milk-derived bacterial isolates modulated intestinal epithelial barrier integrity and cytokine production in vitro, highlighting human milk as a viable source of microbes. Alternatively, fecal material–derived communities may be cocultivated with components of the neonatal diet (61) to help identify bacterial growth requirements derived from the diet or from microbial cross-feeding (62), allowing the prediction of communities likely to thrive in the newborn intestine. In addition to human milk, other bacterial community sources outside of the colon, such as the small intestine, should be considered. Despite limitations inherent to the invasive nature of sampling the small bowel, these microbes might be especially relevant to the newborn given that NEC pathogenesis most often originates in the ileum (63).

As with probiotic selection, it is essential to determine which physiological processes to target to improve health outcomes. Synthetic bacterial communities designed to transform liver-derived primary conjugated bile acids into a diverse array of secondary bile acids (64) could be explored as a means of optimizing digestion, absorption, and assimilation of long-chain fat components of the human milk diet to support postnatal growth. Yet another consideration is colonization resistance, a function of the microbiome that protects against invasion by new (and potentially pathogenic) microbes; tied to this is the consideration of which pathobionts should be targeted. Although the organisms typically associated with sepsis and NEC (e.g., Enterobacteriaceae, *Clostridium perfringens*, and others) seem like obvious choices, comparative analysis of 272 *C*. *perfringens* genomes revealed some strains with hypovirulent properties (65) that could confer beneficial functions (8) and potentially exclude closely related pathogenic strains. Clearly, strain differences must be regarded carefully when considering the strategy of competitive exclusion via synthetic microbial communities. A better understanding of key functions that need to be provided by community replacement will be essential to improving outcomes.

## Future strategy 3: phage therapy

Bacteriophages (phages) are an abundant and crucial modulator of the intestinal microbiota. Fecal material contains 10^9^ virus-like particles per gram, and the vast majority of identified viruses in the human intestine are phage (66, 67). Infants are born without a detectable gut virome but develop one as early as 1 week after birth (68). Gut virome alterations have been associated with numerous diseases, including IBD, colorectal cancer, and NEC (69). A study comparing gut viromes of preterm infants with NEC and a control population of gestational age–matched infants without NEC found that NEC is preceded by reduced viral β-diversity and the occurrence of viral signatures associated with specific bacterial genera (70).

Phage therapy is the use of lytic phage to treat bacterial infections. Discovered in the early 1900s, phages were initially used to treat a variety of infections but fell out of use in Western countries after the discovery of antibiotics (71). The rise of antimicrobial resistance, however, has led to renewed interest in phage therapy. Additionally, the high specificity of phages for their hosts has spurred interest in using phage-based approaches to precisely modulate the microbiome.

**Figure 3:**
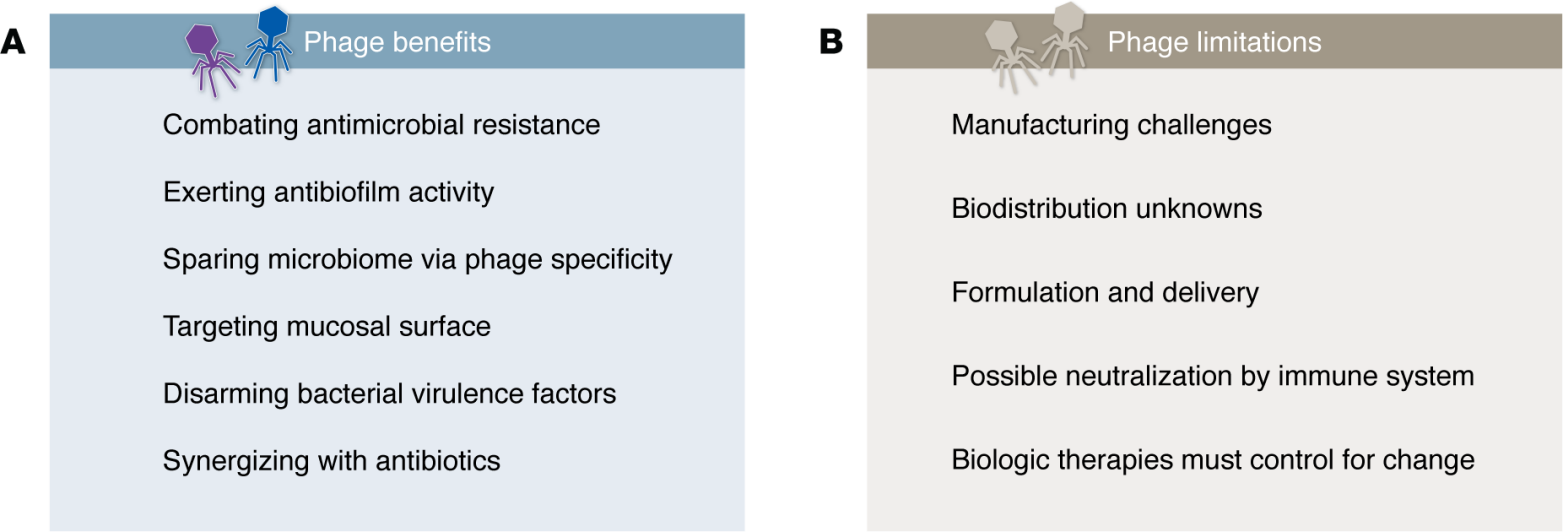
Pros and cons of phage therapy. (**A**) Potential benefits of phage therapy and (**B**) current limitations to clinical applications.

Phage therapy has several advantages over antibiotics (Figure 3). Phages are diverse and ubiquitous — more abundant than known stars in the universe — thus providing a near-infinite supply of therapeutics (72). Should bacterial resistance arise, phages can evolve in response (73). Additionally, bacterial resistance to phages frequently confers substantial fitness costs, such as reduced virulence and resensitization to the complement system (73–75). Some phage-antibiotic combinations interact synergistically, decreasing the amount of antibiotic needed and/or preventing bacterial resistance (76, 77). Phages offer enhanced functionality over antibiotics that contend directly with bacterial challenges, including the ability to destroy biofilms and localize to areas of the mucosal surface where bacteria live (78–81). Phages are considered GRAS (generally regarded as safe) by the FDA; and they have specificity for only their target species and thus are microbiome sparing (71, 82). There are currently no FDA-approved phage therapies, but compassionate use cases have shown promising results, and biotechnology has now entered this space, with several phase II/III trials ongoing (83–86). Phages have also been administered orally to newborns, with no adverse effects (87). In treatment of newborns, phages could avoid selection and proliferation of antimicrobial-resistant pathogens and preclude concerns of increased NEC risk from antibiotic use. Phage therapy has been used against pathogens associated with NEC and neonatal sepsis, including *Escherichia coli*, *Pseudomonas aeruginosa*, *Staphylococcus aureus*, and *Klebsiella pneumoniae* (88–91).

Multiple studies provide evidence that phage-based strategies could modulate the microbiome to prevent NEC. In one study, Brunse et al. prophylactically carried out fecal filtrate transfer (FFT), which excluded bacteria but included phages, in a preterm piglet model (92). The authors compared oral administration of FFT, rectal administration of FFT, and rectal administration of fecal microbiota transplant (FMT) with administration of a saline control. They found that both FFT methods reduced NEC lesion severity, and oral FFT reduced NEC incidence from more than 50% to 0%. Metagenomic and 16S rRNA gene amplicon sequencing identified phages and a small number of eukaryotic viruses in FFT-treated groups, along with alterations in bacterial community composition. While FMT altered intestinal tissue gene expression — including increased expression of lipopolysaccharide response genes — FFT had no such effects. Overall, this study indicates that transfer of phage from a healthy gastrointestinal tract may prevent NEC incidence and reduce severity. However, it does not rule out effects from non-phage components such as bacterial metabolites, and FFT may carry the risk of transferring eukaryotic viruses. The study provides strong support for further investigation of phage-based approaches that prophylactically target the microbiome.

A study by Hsu et al. investigated phage-based microbiota targeting in a mouse model (93). Germ-free mice were inoculated with a model microbiota of ten commensal species that colonize the human gut. On days 16 and 30, mice were orally administered four lytic phages (two each time) that each target a bacterial species. Phages remained at detectable levels for the duration of the experiment (day 44) and reduced, but did not fully eliminate, their bacterial hosts. Additionally, phage activity created a cascading effect on nontargeted bacterial species. While this model does not reflect the full complexity of the human microbiota and is not representative of the neonate gut microbiota, it provides strong evidence that phages can selectively target specific bacterial species, resulting in lasting changes.

Phage therapy could also be used to treat or prevent NEC by targeting pathogens outside of the infant gut. For instance, administering phages to at-risk expectant mothers could reduce the likelihood of NEC and preterm birth while minimizing antibiotic usage. Chorioamnionitis is a risk factor for NEC (94). Additionally, nosocomial outbreaks have been linked to some NEC cases, indicating that pathogen transmission can lead to NEC (94, 95). Ideally, phages would be used prophylactically or at NEC onset. However, compassionate use cases involving bacteremia and sepsis have shown favorable outcomes (84).

**Figure 4:**
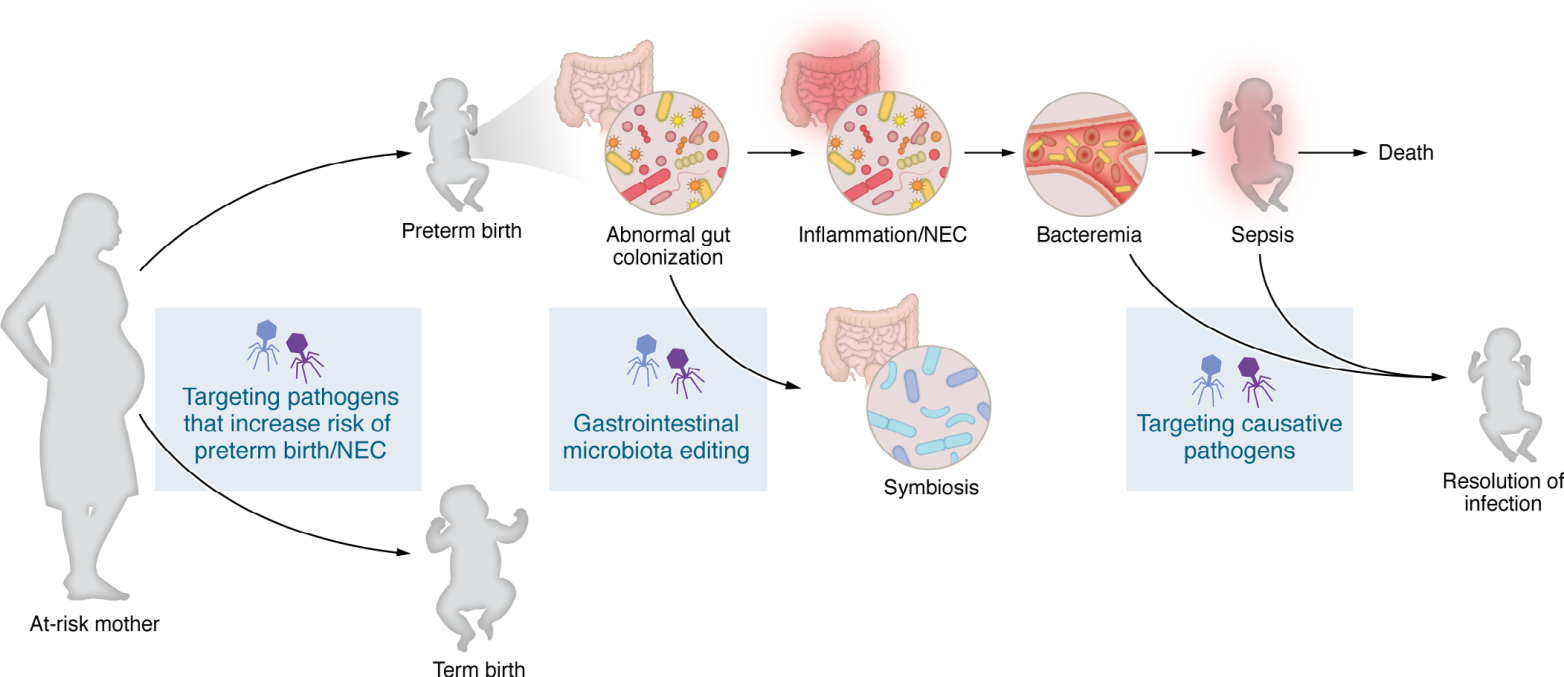
Conceptual framework for phage therapy to treat and prevent NEC. (**A**) Progression of NEC, highlighting points of potential intervention by phage therapy. Preterm birth in at-risk mothers can lead to gut microbiota alterations and inflammation, which may progress to NEC, bacteremia, sepsis, and death. Phage therapy offers a targeted approach to reduce the risk of preterm birth and NEC by targeting pathogenic organisms, restoring gastrointestinal microbial communities, and resolving infections.

While phage therapy shows promise for treating and preventing NEC, challenges remain. Phage therapy is currently a type of personalized medicine in which treatments are formulated for individual patients. The science around phage manufacturing (to be consistent and cost effective), formulation (to increase efficacy and stability), and route of delivery is still being explored (Figure 4). To treat NEC, phage cocktails need to be off-the-shelf and have high efficacy against a wide range of strains. Lack of a single causative agent of NEC presents additional challenges. One solution is the formulation of multiple phage cocktails that target different bacterial species. Several companies are developing and testing off-the-shelf phage treatments, but none specifically aim to prevent NEC (96–98).

Additional knowledge gaps must be addressed. Orally administered phages can travel through the gastrointestinal tract, but factors such as pH and mucin alter phage activity (80, 99). Compared with adults, newborns have substantial differences in their developing immune system and intestine — including decreased gastric acid, mucus production, and polymeric IgA — that would alter phage efficacy (100). Additionally, the innate immune response both aids in bacterial clearance and can neutralize phage particles (99, 101). Phage-immune interactions will likely differ in newborns, making it essential to determine how these factors alter phage efficacy. Another concern is endotoxin release via bacterial cell lysis, which stimulates TLR4. Given that TLR4 is implicated in NEC development, infants could be particularly sensitive to phage-induced endotoxin release. Antibiotics also induce endotoxin release at varying levels (102), but it is unknown whether phage-induced endotoxin release increases NEC risk and severity. Addressing these research gaps will guide the use of phage treatments to maximize benefit and minimize risk.

Finally, while many phage therapy cases have had favorable results, some treatments failed to improve patient outcomes, and the reasons are not fully understood (103, 104). Well-designed RCTs are needed to better understand the efficacy of phage therapy and gain FDA approval of preformulated cocktails. Multiple trials are currently recruiting or underway that should provide insight within the next few years (96, 97, 103, 105).

## When will microbiome-targeting therapies be ready for routine use for newborns?

While currently available probiotic formulations, synthetic bacterial communities, or phage therapy are not yet ready for integration into care of the newborn, key knowledge gaps must be addressed. First, we must achieve a greater understanding of interactions between the preterm infant gut and its microbiome, given that these interactions differ markedly from those in the adult gut. Preclinical models that more accurately represent preterm intestinal physiology can be leveraged for physiologic and therapeutic testing. Recent methodological advances include the cultivation of preterm infant–derived organoids (106, 107) and microfluidic organoid-on-chip models that contain these organoids. Machine learning algorithms that predict NEC (108) and sepsis (109) are being used to predict the risk of translocation and other safety considerations of microbiota-targeting therapeutics. Better animal models are being developed. Piglets can be delivered prematurely via cesarean section and given parenteral nutrition, while germ-free mice allow for the study of humanized microbiomes derived from preterm infants. These improved models will help elucidate unique preterm neonatal gut microbiome-host interactions, key microbial functions that are essential for neonatal health, and safe and effective delivery methods to achieve sustained benefits.

Another fundamental knowledge gap is identifying which infants should be treated with microbiome-targeting therapies. No microbial or host biomarker accurately predicts which infants will develop NEC or sepsis, and although nearly all preterm infants have an altered gut microbiome relative to healthy term infants, the majority do not develop comorbid disease. An argument can be made to therapeutically target all newborns undergoing microbiota-disrupting procedures or medications. Given the low complexity of newborn gut microbial communities, impacts on the infant relative to the adult gut likely are greater, and recovery more prolonged. Disruptions during the critical developmental window representing the first 1,000 days of life could mediate the association between early-life antibiotic exposure and chronic disease (110). Indeed, microbiota disruptions are associated with increased risk of autoimmune diseases, allergies, IBDs, and neurodevelopmental disorders (111). While pursuing a deeper understanding of how gut microbiota interventions might reduce the risk of death and devastating disease in the neonatal period, the improved preclinical models can be leveraged to gain insights into mechanisms by which these chronic diseases occur. Ultimately, microbiome-targeting therapies in the newborn period have the potential to alleviate allergic, metabolic, inflammatory, and cognitive disorders later in life.

Future gut microbiota restoration strategies are not limited to probiotics, synthetic bacterial communities, and phage therapy. If beneficial microbial functions and specific strains conferring those functions to the neonatal gut can be identified, targeted prebiotics might enhance the growth or functions of those species. Vaginal seeding and maternal FMT are being tested for microbiome restoration following antibiotic exposure. Dietary strategies including sustained exclusive breastfeeding can promote the development of gut microbial communities (112).

As our understanding of complex microbe-host interactions in the preterm neonatal gut advances and clinically relevant models for assessing safety and efficacy are developed, microbiome-targeting therapeutics may emerge as a promising strategy to improve health outcomes in this vulnerable population. Well-designed clinical trials, improved neonatal models, and a deeper understanding of host-microbiome interactions will be critical in translating these therapies into clinical practice. Addressing these challenges will determine whether microbiome-targeting therapeutics can fulfill their promise to prevent both acute and chronic disease across the lifespan.

## Version 1 06/02/2025

Electronic publication
